# Supplementary material for: MetaRibo-Seq measures translation in microbiomes
Source: Nat Commun. 2020 Jun 29;11:3268. doi: 10.1038/s41467-020-17081-z (PMC7324362; doi:10.1038/s41467-020-17081-z)
Supplement: Supplementary file 10 — Supplementary Data 7 [file 41467_2020_17081_MOESM10_ESM.zip › File2/Confidence_High_Taxonomy/132208_out.krona.html]

Javascript must be enabled to view this page.

members
magnitude
magnitudeUnassigned
count
unassigned
taxon
rank

132208\_out

7

superkingdom
2
7

1239
5
phylum

1263017

SRS012969\_contig\_number\_31503SRS017307\_contig\_number\_contig-100\_12845.104290SRS055017\_contig\_number\_34904SRS077392\_contig\_number\_35724SRS077849\_contig\_number\_contig-100\_1322.167321
5
species

544448
2
phylum

2
31969
class

186329
2
order

2

SRS011134\_contig\_number\_18369SRS098571\_contig\_number\_6277
2053494
species
